# Rhabdomyolysis Secondary to COVID-19 Vaccination

**DOI:** 10.7759/cureus.15004

**Published:** 2021-05-13

**Authors:** Mariah Mack, Laura Nichols, Dubert M Guerrero

**Affiliations:** 1 Internal Medicine, University of North Dakota School of Medicine and Health Sciences, Fargo, USA; 2 Internal Medicine, Sanford Health, Fargo, USA; 3 Infectious Diseases, Sanford Health, Fargo, USA

**Keywords:** covid-19, vaccine, sars-cov-2 vaccination, non-traumatic rhabdomyolysis, myalgia

## Abstract

Rhabdomyolysis has been described as a complication of coronavirus disease 2019 (COVID-19) infection, but few cases of rhabdomyolysis associated with COVID-19 vaccination have been reported. We described a case of an 80-year-old male who developed rhabdomyolysis two days after receiving his second dose of the Moderna COVID-19 vaccine. He presented with severe weakness, myalgias, and an initial creatinine kinase (CK) of 6,546 IU/L that improved with intravenous fluids. Common causes of rhabdomyolysis were excluded including statin use, strenuous exercise, and trauma. With the increasing immunization efforts against COVID-19, physicians should consider the possibility of rhabdomyolysis when a patient presents with neuromuscular complaints following vaccination.

## Introduction

Common causes of rhabdomyolysis include immobilization, strenuous exercise, and medications. Rhabdomyolysis can also be caused by infections including HIV, coxsackievirus, influenza A and B, and several bacterial infections [1]. Acute viral infections have been shown to be directly myotoxic, but toxicity may also be caused by associated hyperthermia and dehydration [1]. Rhabdomyolysis has also been established as a complication of coronavirus disease 2019 (COVID-19) infection [2-5]. More recently, one case of rhabdomyolysis following Oxford/AstraZeneca COVID-19 vaccination was described in a patient with carnitine palmitoyltransferase II (CPT II) deficiency [6]. We describe one of the first cases of rhabdomyolysis following Moderna vaccination for COVID-19 in a patient without predisposing factors.

## Case presentation

An 80-year-old man with a history of type II diabetes mellitus presented to the ER via ambulance with the chief complaint of generalized body aches and acute difficulty with ambulation. The patient received his second Moderna COVID-19 vaccine two days prior to admission. The next day he developed generalized body aches, nausea, and an episode of vomiting. He denied any fevers or chills. Though he normally ambulates independently, he was unable to stand or get out of bed due to weakness and generalized pain when he woke up the morning of admission. His admission medications included alogliptin, empagliflozin, insulin aspart, insulin glargine, losartan, metformin, pioglitazone, and tamsulosin. He reports no recent changes in medications or intake of over-the-counter medications. Past medical history was significant for COVID-19 infection three months prior, which required hospitalization for 13 days and treatment with convalescent plasma, remdesivir, and dexamethasone, according to institutional standard treatment protocols at the time.

On exam, vital signs were stable with a blood pressure of 120/70 mmHg, heart rate of 87/minute, temperature of 98.4° F, and respiratory rate of 16/minute. He was in no acute distress. His strength was normal and symmetric bilaterally, cranial nerves II-XII were grossly intact, and sensory was intact to light touch bilaterally. An electrocardiogram (EKG) was negative for ischemia. Troponins, a respiratory polymerase chain reaction (PCR) panel including influenza, and COVID-19 PCR were negative. Initial laboratory findings are summarized below (Table 1). His serum creatinine kinase (CK) was 6,546 U/L. Complete metabolic panel revealed potassium 3.6 meq/L, sodium 130 meq/L, chloride 96 meq/L, glucose 219 mg/dL, blood urea nitrogen (BUN) 23 mg/dL, creatinine 1.1 mg/dL, aspartate aminotransferase (AST) 112 U/L, and alanine aminotransferase (ALT) 47 U/L. His inflammatory markers were elevated with lactate dehydrogenase (LDH) 359 U/L, erythrocyte sedimentation rate (ESR) 52 mm/Hr, and C-reactive protein (CRP) 146.8 mg/dL. Urinalysis excluded myoglobinuria and creatinine remained in his baseline range.

**Table 1:** Laboratory findings at admission. ALP: Alkaline phosphatase; ALT: Alanine aminotransferase; AST: Aspartate aminotransferase; BUN: Blood urea nitrogen; CK: Creatine kinase; CRP: C-reactive protein; ESR: Erythrocyte sedimentation rate; LDH: Lactate dehydrogenase; RBC: Red blood cell; WBC: White blood cell.

	Admission	Reference range
Hemoglobin	13.3	13-15 g/dL
RBC	4.6	4.6-6.8 x 106/mcL
WBC	6.2	3.6-10.3 x 103/mcL
Platelet	145	140-420 x 103/mcL
Sodium	130	135-145 mmol/L
Potassium	3.6	3.7-5.1 mmol/L
CK	6,546	30-200 U/L
Glucose	219	70-100 mg/dL
BUN	23	6-24 mg/dL
Creatinine	1.1	0.6-1.3 mg/dL
Calcium	8.5	8.5-10.5 mg/dL
Chloride	96	99-110 meq/L
Bilirubin total	0.8	0.2-1.2 mg/dL
ALP	66	30-150 U/L
ALT	47	0-35 U/L
AST	112	0-35 U/L
CRP	147	0.0-8.0 mg/L
ESR	52	0-19mm/Hr
LDH	359	125-245 u/L

Rhabdomyolysis was diagnosed based on acute muscular pain and elevated CK. He is a diabetic elderly who had some nausea and vomiting but common causes of rhabdomyolysis were excluded as the patient denied trauma, recent surgery, strenuous exercise, alcohol use, or illicit drug use. He was not taking medications known to cause rhabdomyolysis including statins. No infections were identified. His serum glucose level was not elevated to a level that would point to a hyperosmolar hyperglycemic state.

Given the timeline of events, lack of alternate explanation, and known correlation of COVID-19 and rhabdomyolysis, the patient was felt to have vaccine-related rhabdomyolysis. The Naranjo score is a causality assessment tool validated to determine the likelihood of an adverse drug reaction [7]. For this patient, positive findings included the adverse event that appeared after the drug was given, the adverse event improved after the drug was discontinued, alternative causes were ruled out, and objective evidence of the adverse event was available (CK). The Naranjo Score was 6, which indicates the rhabdomyolysis was probably an adverse reaction to vaccination.

He received IV fluids overnight. His CK improved over the course of his hospital stay, and he did not develop significant acute kidney injury or electrolyte abnormalities. The patient regained his ability to ambulate independently, had improved myalgias, and was discharged in stable condition.

## Discussion

Here we describe a case of rhabdomyolysis following COVID-19 vaccination. Due to the short time frame since widespread COVID-19 vaccination efforts began, there is limited published data available about adverse effects in the general public, particularly in patients who were previously diagnosed with COVID-19 as they were excluded from original trials. To the best of our knowledge, there has not been a published case report of rhabdomyolysis following this mRNA COVID-19 vaccination.

Rhabdomyolysis has been described with COVID-19 infection and other viral infections. The mechanism of viral rhabdomyolysis in COVID-19 infection has yet to be established. One possible mechanism is the direct viral invasion of myocytes [5]. However, to date, the COVID-19 virus has not been detected in skeletal muscle [8]. The host immune response to viral infection could also cause muscle injury. This could occur due to an exaggerated immunological reaction resulting in a “cytokine storm” - like immune response [5]. Further research is needed to determine if the proposed mechanisms of rhabdomyolysis in COVID-19 infection may contribute to rhabdomyolysis following COVID-19 vaccination.

Several reports of rhabdomyolysis secondary to other vaccinations have also been reported, most commonly to influenza vaccination [9, 10]. A potential mechanism of rhabdomyolysis after vaccination is an exaggerated immune response to adjuvants. This has been previously described as autoimmune/inflammatory syndrome induced by adjuvants (ASIA), which is the spectrum of autoimmune phenomena that are induced following the exposure to external factors with adjuvant activity [11]. It can lead to inflammatory sequela such as rhabdomyolysis. In this case report, our patient received the vaccine despite a prior history of COVID-19 infection. It is unclear, yet possible that the previous infection affected his reaction to the vaccination.

An mRNA vaccine is a new type of COVID-19 vaccine. RNA stimulates the immune system, and therefore acts as an adjuvant by activating specific toll-like receptors [12]. In addition, previous exposure to the COVID-19 virus may potentiate an increased immune response to the vaccine. In one study, after one dose of the Pfizer/BioNTech vaccine, participants who had prior COVID-19 infection showed antibody responses 6.8 times higher and T-cell responses 5.9 times higher than those without prior infection [13]. This increased immune response in patients with prior COVID-19 infection may have contributed to our patient’s reaction to the vaccination. Rates of fatigue, myalgias, and arthralgias following Moderna vaccination in phase three trials were 70.0%, 61.5%, and 46.4% respectively [14]. Myositis in the deltoid has been described after the COVID-19 vaccination [15]. More generalized myositis could progress to rhabdomyolysis as in our patient.

A case of rhabdomyolysis following Oxford/AstraZeneca COVID-19 vaccination was recently described in a patient with CPT II deficiency [6]. The 34-year-old man presented five hours after vaccination with fever, myalgia, weakness, and hematuria. His CK peaked at 250,000 U/l on day three. He did not have a prior COVID-19 infection. Neuromuscular disorders such as CPT II deficiency can predispose individuals to develop rhabdomyolysis. The patient in our case had no known neuromuscular disorders. In addition, the Oxford/AstraZeneca vaccine is an adenoviral vector vaccine, while in our case, our patient received an mRNA vaccine.

A search of the Vaccine Adverse Event Reporting System (VAERS) database on April 24, 2021, yielded 35 events associated with COVID-19 vaccination and rhabdomyolysis [16]. Many reports involved confounding factors such as statin use, infection, and falls with prolonged downtime. However, many of the reports were similar to our case. These occurred after any mRNA vaccination, in both men and women, ages 23-84, and the peak CK ranged from 1,300 - 41,000 U/L. One report involved an 83-year-old female on simvastatin who received the second dose of her COVID-19 vaccine. That evening, she began to have diffuse myalgias, became too weak to get up from a seated position, and could not walk. She was admitted to the hospital for rhabdomyolysis. Her CK was 9,678, AST 176, ALT 55, and she had myoglobinuria. She was hydrated and did not develop acute kidney injury.

A second report involved a 48-year-old female who received the second dose of vaccine [16]. Seven days later, she developed myalgias, difficulty walking, and gross hematuria. She was admitted for rhabdomyolysis, and her CK was 41,000. She was not taking any statins. She was hospitalized for four days and has recovered. Although these cases required hospitalization, patients with mild rhabdomyolysis may not seek medical care and the incidence may be underreported. Clinicians should be aware of the potential for rhabdomyolysis following COVID-19 vaccination that may require early intervention.

## Conclusions

Given the benefits of the COVID-19 vaccine, we encourage ongoing immunization efforts. However, physicians should consider the possibility of rhabdomyolysis when a patient presents with neuromuscular complaints following COVID-19 vaccination. Early recognition and treatment are essential to avoid acute kidney injury. Ongoing surveillance is required to evaluate the incidence of rhabdomyolysis following COVID-19 vaccination, especially in patients who were previously infected with COVID-19.

## References

[1] Khan SF (2018). Rhabdomyolysis. Current diagnosis & treatment: nephrology & hypertension.

[2] Jin M, Tong Q (2020). Rhabdomyolysis as potential late complication associated with COVID-19. Emerg Infect Dis.

[3] Singh B, Kaur P, Reid RR (2020). Case reports: rhabdomyolysis associated with COVID-19. Am Fam Physician.

[4] Taxbro K, Kahlow H, Wulcan H, Fornarve A (2020). Rhabdomyolysis and acute kidney injury in severe COVID-19 infection. BMJ Case Rep.

[5] Haroun MW, Dieiev V, Kang J (2021). Rhabdomyolysis in COVID-19 patients: a retrospective observational study. Cureus.

[6] Tan A, Stepien KM, Narayana ST (2021). Carnitine palmitoyltransferase II deficiency and post-COVID vaccination rhabdomyolysis. QJM Int J Med.

[7] Naranjo CA, Busto U, Sellers EM (1981). A method for estimating the probability of adverse drug reactions. Clin Pharmacol Ther.

[8] Ding Y, He L, Zhang Q (2004). Organ distribution of severe acute respiratory syndrome (SARS) associated coronavirus (SARS-CoV) in SARS patients: implications for pathogenesis and virus transmission pathways. J Pathol.

[9] Callado RB, Carneiro TG, Parahyba CC, Lima NA, da Silva Junior GB, Daher EF (2013). Rhabdomyolysis secondary to influenza A H1N1 vaccine resulting in acute kidney injury. Travel Med Infect Dis.

[10] Rajaratnam N, Govil S, Patel R, Ahmed M, Elias S (2021). Rhabdomyolysis after recombinant zoster vaccination: a rare adverse reaction. J Community Hosp Intern Med Perspect.

[11] Shoenfeld Y, Agmon-Levin N (2011). 'ASIA' - autoimmune/inflammatory syndrome induced by adjuvants. J Autoimmun.

[12] Dalpke AH, Helm M (2012). RNA mediated Toll-like receptor stimulation in health and disease. RNA Biol.

[13] Manisty C, Otter AD, Treibel TA (2021). Antibody response to first BNT162b2 dose in previously SARS-CoV-2-infected individuals. Lancet.

[14] Baden LR, El Sahly HM, Essink B (2021). Efficacy and safety of the mRNA-1273 SARS-CoV-2 vaccine. N Engl J Med.

[15] Theodorou DJ, Theodorou SJ, Axiotis A, Gianniki M, Tsifetaki N (2021). COVID-19 vaccine-related myositis. QJM Int J Med.

[16] (2021). United States Department of Health and Human Services (DHHS), Public Health Service (PHS), Centers for Disease Control (CDC) / Food and Drug Administration (FDA), Vaccine Adverse Event Reporting System (VAERS) 1990 - 4/16/2021, CDC WONDER On-line Database. https://wonder.cdc.gov/vaers.html.

